# Lebein, a Snake Venom Disintegrin, Induces Apoptosis in Human Melanoma Cells

**DOI:** 10.3390/toxins8070206

**Published:** 2016-07-05

**Authors:** Manel B. Hammouda, María F. Montenegro, Luis Sánchez-del-Campo, Ons Zakraoui, Zohra Aloui, Ichrak Riahi-Chebbi, Habib Karoui, José Neptuno Rodríguez-López, Khadija Essafi-Benkhadir

**Affiliations:** 1 Laboratoire d’Epidémiologie Moléculaire et Pathologie Expérimentale Appliquée Aux Maladies Infectieuses (LR11IPT04), Institut Pasteur de Tunis, 1002 Tunis, Tunisia; ma_nel85@hotmail.com (M.B.H.); zakraoui-ons@hotmail.fr (O.Z.); zohra.aloui@pasteur.rns.tn (Z.A.); ichrakriahi@live.com (I.R.-C.); habib.karoui@pasteur.rns.tn (H.K.); 2 Université de Tunis El Manar, 1068 Tunis, Tunisia; 3 Department of Biochemistry and Molecular Biology A, School of Biology, University of Murcia, 30100 Espinardo, Murcia, Spain; fermontenegro@um.es (M.F.M.); sancampo@um.es (L.S.-d.-C.); 4 Instituto Murciano de Investigación Biosanitaria (IMIB), 30120 Murcia, Spain

**Keywords:** Lebein, disintegrin, snake venom, *Macrovipera lebetina*, melanoma, apoptosis

## Abstract

Melanoma, the most threatening form of skin cancer, has a very poor prognosis and is characterized by its very invasive and chemoresistant properties. Despite the recent promising news from the field of immunotherapy, there is an urgent need for new therapeutic approaches that are free of resistance mechanisms and side effects. Anti-neoplasic properties have been highlighted for different disintegrins from snake venom including Lebein; however, the exact effect of Lebein on melanoma has not yet been defined. In this study, we showed that Lebein blocks melanoma cell proliferation and induces a more differentiated phenotype with inhibition of extracellular signal-regulated kinase (ERK) phosphorylation and microphthalmia-associated transcription factor (MITF) overexpression. Melanoma cells became detached but were less invasive with upregulation of E-cadherin after Lebein exposure. Lebein induced a caspase-independent apoptotic program with apoptosis inducing factor (AIF), BCL-2-associated X protein (BAX) and Bim overexpression together with downregulation of B-cell lymphoma-2 (BCL-2). It generated a distinct response in reactive oxygen species (ROS) generation and p53 levels depending on the p53 cell line status (wild type or mutant). Therefore, we propose Lebein as a new candidate for development of potential therapies for melanoma.

## 1. Introduction

Melanoma is the most aggressive form of skin cancer, with an increasing incidence in the last 30 years and with an intrinsic metastatic and chemoresistance potential that is responsible for its poor prognosis and survival after diagnosis [1,2]. The approval of new therapeutic strategies has changed the scenario over the last few years. The use of BRAF-MEK small molecule inhibitors, together with the development of antitumour immune response promoters (CTLA4 and PD-1/PD-L1), has increased positive patient outcomes and survival rates [3]. However, due to different acquired resistance mechanisms, recurrence and toxic side effects, it is desirable to explore novel therapeutics approaches.

Natural compounds from different sources such as plants, microorganisms and marine organisms are currently being used in clinics for the treatment of many diseases and, importantly, for cancer treatment. Many compounds from venomous animals, such as frogs, spiders, bees, scorpions, caterpillars, insects, wasps, centipedes, ants, toads and snakes, have shown new biotechnological and pharmacological applications [4]. Disintegrins constitute a family of low molecular weight proteins from viper venoms that specifically bind cell surface integrins on different cell types, including tumour cells, and they act as competitive inhibitors. Integrins, are important surface adhesion molecules and cell signalling receptors that regulate cell proliferation, migration and cell survival [5,6]. Due to this important role, disintegrins have been proposed as potential cancer therapy molecules targeting cell-to-cell and cell-to-matrix interactions with effects on key cancer progression, metastasis and processes such as cancer cell motility, invasion and proliferation [7,8,9]. For this reason, we evaluated the effect of the disintegrin Lebein, which was isolated from *Macrovipera lebetina*, on the human melanoma cell lines SK-MEL-28 and LU-1205. Lebein showed antiproliferative activity through extracellular signal-regulated kinase (ERK) phosphorylation inhibition and the microphthalmia-associated transcription factor (MITF) up-regulation, invasiveness suppression by E-cadherin overexpression, loss of cell adhesion and caspase-independent apoptosis program activation. In this report, we propose the disintegrin Lebein as a novel potential therapeutic agent for melanoma.

## 2. Results

### 2.1. Lebein Decreases Cell Viability and Induces Apoptosis in Melanoma Cells

**Figure 1:**
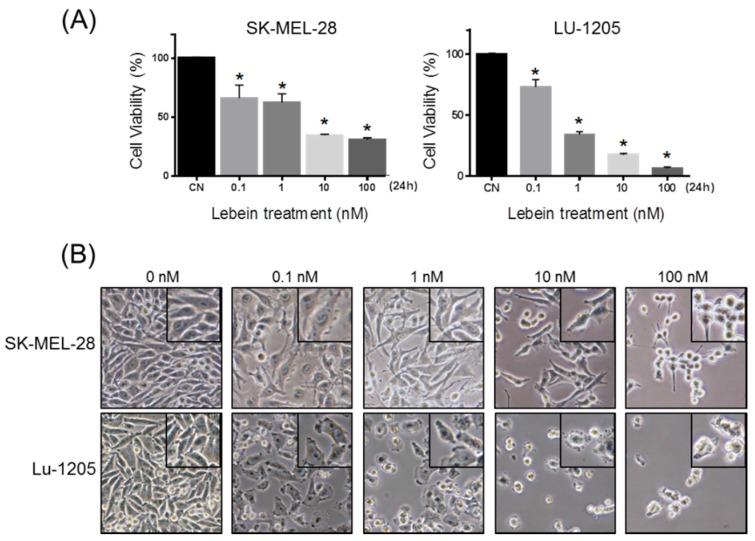
Lebein inhibits cell viability. (**A**) Melanoma cells SK-MEL-28 and LU-1205 were treated with 0, 0.1, 1, 10 and 100 nM of Lebein for 24 h. Cell viability was determined using an MTT assay and by measuring the absorbance at 490 nm. Values were normalized to untreated cells (CN) and are expressed as the mean ± SD. Assays were performed in triplicate. * *p* < 0.05 with respect to CN; (**B**) The effects of Lebein on SK-MEL-28 and LU-1205 cell morphology. Cells were treated with increasing concentrations of Lebein, and photos were taken after 24 h.

Different disintegrins have been shown to possess antiproliferative activity for a variety of tumour cells [9,10,11,12]. We have previously demonstrated that Lebein, an heterodimeric disintegrin isolated from *Macrovipera lebetina* snake venom, inhibits colon tumour growth in vivo [9]. Here, we investigated the antiproliferative effect of Lebein on SK-MEL-28 and LU-1205 melanoma cells. The cells were treated with different concentrations of Lebein (0.1 nM to 100 nM), and cell viability was evaluated with an MTT assay after 24 h (Figure 1A). With respect to vehicle treated controls, Lebein significantly decreased the viability of SK-MEL-28 and LU-1205 cells (Figure 1A). Importantly, this inhibition was dose dependent, with the inhibition increasing at higher concentrations of Lebein.

**Figure 2:**
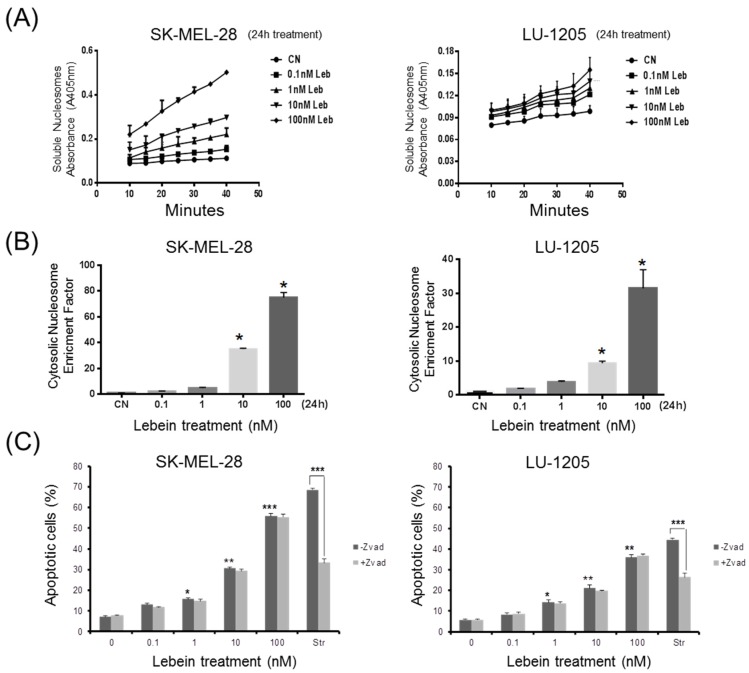
Lebein induced apoptotic cell death in SK-MEL-28 and LU-1205 melanoma cells. (**A**) Measure of the absorbance at 405 nm from the soluble nucleosomes; (**B**) The cytosolic nucleosome enrichment factor was determined after 24 h of treatment as explained in the Material and Methods section; (**C**) Flow cytometry analysis using Annexin-V/7-AAD staining of Z-VAD-fmk (20 µM)-pretreated melanoma cells cultured in the absence (control) and the presence of Lebein for 24 h. Staurosporine (2 µM, Str) was used as a positive control of apoptosis. * *p* < 0.05; ** *p* < 0.01 and *** *p* < 0.005 with respect to untreated controls.

Melanoma cells treated with Lebein showed morphological changes such as a loss of anchorage, reduction in volume, rounded appearance, chromatin condensation and blebbing (Figure 1B). Because both proliferation inhibition and morphological changes after Lebein treatment are compatible with cell death, different experiments were designed to elucidate the type of cell death observed. An important biochemical hallmark of apoptosis is the detection of fragments of genomic DNA (mono- and oligonucleosomes) in the cytoplasm of apoptotic cells [13]. Induction of apoptosis was analysed in SK-MEL-28 and LU-1205 cells after Lebein treatment using a combination of anti-histone and anti-DNA capture in an ELISA method with absorbance measurement. Melanoma cells were incubated for 24 h with different concentrations of Lebein (from 0.1 to 100 nM), and the presence in the cytoplasm of free nucleosomes (mono- and oligonucleosomes) was evaluated and shown to be an enrichment factor, which is indicative of apoptotic activity (Figure 2A,B). Significant increases in nucleosome fragments after 24 h were observed in both cell lines at 1, 10 and 100 nM compared to the corresponding vehicle-treated cells. Thus, these results indicated that Lebein induces apoptotic cell death in SK-MEL-28 and LU-1205 melanoma cells.

To determine the role of caspase activation in Lebein-induced apoptosis, SK-MEL-28 and LU-1205 melanoma cell lines were treated with the pan-caspase inhibitor, z-VAD-fmk (20 µM), 2 h before adding Lebein at different concentrations (0.1 nM to 100 nM for a further 24 h). The percentage of apoptotic cells was quantified by flow cytometry after Annexin-V staining. Our results indicated that the inhibition of caspases did not prevent the apoptotic effect of Lebein (Figure 2C), suggesting that the effect of Lebein in melanoma cells was independent of caspase activation.

### 2.2. Lebein Modulates ROS Generation in Melanoma Cells

**Figure 3:**
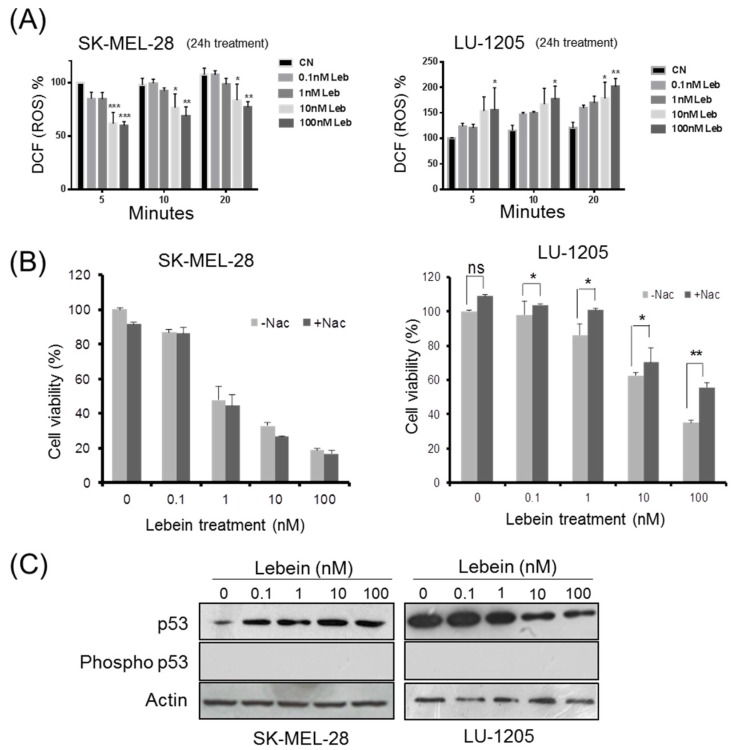
Effect of Lebein on reactive oxygen species (ROS) generation and p53 expression. (**A**) The production of ROS after 24 h of Lebein exposure was evaluated by detection of fluorescence using the fluorogenic probe CM-H2DCFDA at 5, 10 and 20 min. The results were normalized to control untreated cells (CN). * *p* < 0.05; ** *p* < 0.01 and *** *p* < 0.005 when compared to their respective CN; (**B**) Cells were pre-treated with NAC (1 mM) for 2 h before treatment with Lebein (0.1, 1, 10, 100 nM) for 24 h. Cell viability was estimated by MTT assay. Data are representative of at least triplicate experiments. * *p* < 0.05 and ** *p* < 0.005; ns, non-significant; (**C**) Western blot analysis of p53 expression of SK-MEL28 and LU-1205 cells after treatment with Lebein. The total expression levels and activation were evaluated using a p53 and phospho p53 at Ser-15 specific antibodies after 24 h of indicated treatments. Actin was used as a control for equal loading.

Many studies have shown that in some circumstances reactive oxygen species (ROS) generation contributes to the initiation of the apoptotic signalling cascade [14]. One study in particular reported that Vipera toxin venom from the *Lebetina turanica* snake induces apoptosis in colon cancer cells by ROS formation [15]. To better understand the effect of the snake venom Lebein on melanoma cells, we evaluated ROS levels in SK-MEL-28 and LU-1205 melanoma cells treated with different concentrations of Lebein (Figure 3A). Intriguingly, the Lebein ROS formation outcome was different depending on the cell line. Although Lebein stimulated ROS generation in LU-1205 cells, it inhibited the formation of ROS in SK-MEL-28 cells. To further assess the significance of ROS in mediating the Lebein-induced inhibition of cell viability, we used a ROS scavenger, *N*-acetylcysteine (NAC) to lower ROS production in LU-1205 cells. As expected, NAC inhibited Lebein-induced ROS generation (data not shown). Interestingly, on Lebein treatment, NAC partially diminished the inhibitory effect of Lebein on LU1205 cell viability (Figure 3B). At a concentration of 100 nM of Lebein, the percentage of viable cells increased from 35.1% in Lebein treated cells to 53.3% in the presence of NAC showing that ROS generation was in part required for cell viability inhibition in LU-1205 cells.

Despite the surprising result obtained for the SK-MEL-28 cell line, some studies have indicated that elevated levels of intracellular ROS contribute to early events involved in cancer initiation and progression and suggest the use of antioxidants to reduce ROS levels in tumour cells [16]. We propose Lebein as a treatment acting as a pro-oxidant on LU-1205 cells in which intracellular levels of ROS are not important to reach cell toxicity to produce apoptosis. In contrast, SK-MEL-28 Lebein could act as an antioxidant to deplete ROS from tumour cells. Furthermore, another study [17] suggested that there is a complex relationship between p53 and levels of ROS. Thus, it has been proposed that p53 downregulates intracellular levels of ROS in an antioxidant-fashion but can also exert a pro-oxidant function inducing the generation of ROS products that contribute to apoptosis.

### 2.3. Lebein Modifies p53 Levels in a Cell-Specific Way Depending on the p53 Mutation Status in the Melanoma Cells

It has been reported that p53 overexpression only induced apoptosis in the p53-mutated cell lines and not in p53-WT melanoma cells [18], suggesting that overexpression of p53 was not enough to induce apoptosis. In contrast to other solid tumours, melanoma generally lacks p53 mutations and retains the expression of wild-type protein (WT), often at high levels [19,20]. To understand the effect of Lebein on p53 expression in melanoma and the ability of this tumour suppressor gene to induce apoptosis, we developed western blot experiments for mutant-p53 (SK-MEL-28) and WT-p53 (LU-1205) cell extracts after Lebein treatment (Figure 3C). Our results showed an increase in p53 expression in SK-MEL-28 cells and a decrease in the case of the LU-1205 cell line after exposing the cells to increasing concentrations of Lebein. However, we did not observe phosphorylation of p53 at Ser-15 after Lebein treatment in both melanoma cell lines (Figure 3C). This consequently suggested that p53 activation was not necessary to trigger the apoptotic cascade because in both cell lines there was a clear cell death outcome independent of the p53 status. Importantly, it is known that different parameters, such as cell type, the nature of the stress and the intensity of the stimuli, contribute to the outcome of the p53-ROS interaction, limiting any genetic mechanistic generalization [21].

### 2.4. Lebein Induces Downregulation of Cell Survival Kinase ERK in Melanoma Cells

**Figure 4:**
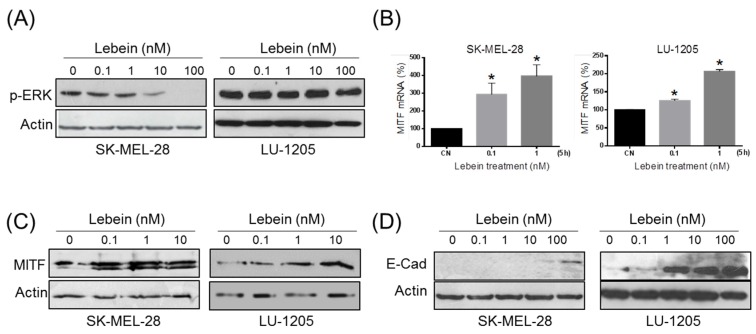
Lebein induced SK-MEL-28 and LU-1205 extracellular signal-regulated kinase (ERK) activation and cell differentiation. (**A**) Analysis of ERK phosphorylation by western blotting using a phospho specific antibody after 24 h of the indicated Lebein treatments. The results are representative of three independent experiments; (**B**) Quantitative real-time PCR of MITF mRNA. SK-MEL-28 and LU-1205 cells were treated with the indicated concentrations of Lebein (5 h). mRNA levels are represented relative to β-actin and were compared with their expression levels in untreated cells (CN). * *p* < 0.05 with respect to CN; (**C**) Total MITF protein expression was evaluated by western blotting after 24 h of Lebein treatment; (**D**) Expression of E-cadherin was studied after 24 h of the indicated treatments with Lebein by WB. The data are representative of three independent experiments.

Melanoma cells are characterized by continuous activation of the MEK-ERK kinase pathway, which is responsible for uncontrolled proliferation due to activating mutations in B-RAF and N-RAS [22]. It is known that ERK plays an important role not only in cell proliferation but also in cell survival, differentiation and ROS generation [23]. Different anti-neoplastic compounds can inhibit the MAP/ERK1/2 pathway at different levels, and they work as antiproliferatives [24] due to inhibition of ERK phosphorylation activation [25]. Therefore, we asked whether Lebein induced cell death was associated with ERK activation. After 24 h of treatment with different concentrations of Lebein, western blot assays showed a reduction in ERK phosphorylation compared to the controls in both SK-MEL-28 and LU-1205 cells (Figure 4A). These data suggested that Lebein antiproliferative and pro-apoptotic effects could be associated with targeting the MEK-ERK kinase pathway.

### 2.5. Lebein Induces a More Differentiated and Less Invasive Phenotype in Melanoma Cells

Many genes involved in different cellular processes that are important for melanoma progression, such as proliferation, migration, invasiveness and senescence, are regulated by MITF, which has been proposed as the melanocytic lineage master regulator [26]. MITF cellular levels need to be tightly regulated to keep cells proliferating because microenviromental changes or the addition of certain drugs can induce a phenotype switch from proliferative to differentiated with no cell division [27,28]. Previous studies have suggested that MITF protein levels are negatively regulated by proteosomal degradation induced by the extracellular signal-regulated kinase pathway [29,30]. Numerous natural compounds have been reported to induce differentiation of melanoma cells through up regulation of MITF due to inhibition of ERK phosphorylation [25]. Because Lebein induced a decrease in ERK-phosphorylation, we examined whether this could have an impact on MITF levels and downstream cellular processes. As expected, this blockage in ERK activity resulted in an up-regulation of MITF mRNA and protein levels (Figure 4B,C). Notably, these results contribute to the explanation of the antiproliferative effect of Lebein on melanoma cells, increasing MITF levels through inhibition of ERK phosphorylation.

It has been shown that loss of E-cadherin but not the disruption of cell-cell contact is able to promote invasiveness [31] and that in melanoma, disruption of cell invasiveness correlates with E-cadherin up-regulation [32]. According to the rheostat model proposed for MITF in melanoma cells [27], increasing levels of MITF are associated not only with a more differentiated phenotype with less cell proliferation, as previously shown, but also with noninvasive properties. Within this context, we evaluated the effect of Lebein on E-cadherin expression in melanoma cells. Western blot assays showed a dose-dependent increase in E-cadherin in both cell lines after 24 h of Lebein treatment (Figure 4D). Therefore, our results indicated that Lebein induced up-regulation of MITF and E-cadherin, which is compatible with a more differentiated and less invasive phenotype.

### 2.6. Lebein Induces Caspase-Independent Cell Death in Melanoma Cells

**Figure 5:**
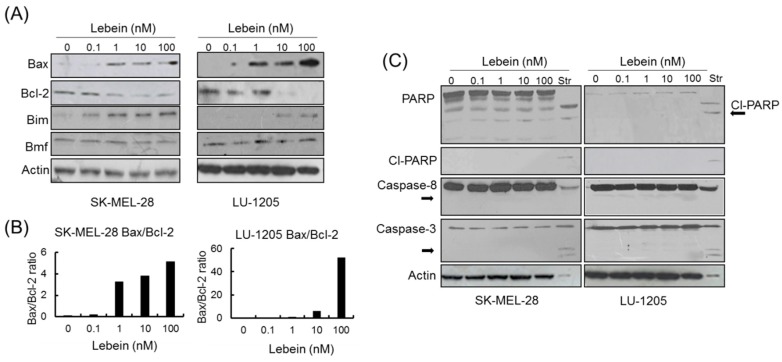
Activation of apoptotic responses in melanoma cells by Lebein. (**A**) SK-MEL-28 and LU-1205 cells were treated with increasing concentrations of Lebein for 24 h. Western blot experiments are presented; (**B**) The ratio between BCL-2-associated X protein (BAX) and B-cell lymphoma-2 (BCL-2) protein expression levels was calculated by densitometry analysis; (**C**) Western blot of several apoptosis-related proteins after Lebein treatment (24 h). Staurosporine (2 µM, Str) was used as a positive control of apoptosis. Cleaved PARP (Cl-PARP). The arrows indicate the molecular weight for activated caspases.

BCL-2 was the first anti-apoptotic gene discovered with clear implications in tumour biology [33]. BAX, on the other hand, was the first identified pro-apoptotic gene, and it encodes a protein that belongs to the BCL-2 family [34]. BAX can form heterodimers with BAK, another member of the BCL-2 family, and these BAX/BAK heterodimers are responsible for releasing cytochrome c from the mitochondria during the apoptotic activation response; whereas the interaction of BCL-2 with BAX/BAK is able to sequester these proteins with anti-apoptotic outcomes [35]. As the BAX/BCL-2 ratio seems to be the determinant for setting a threshold for cells undergoing apoptosis [36], we studied the effect of Lebein on BCL-2 and BAX protein expression to understand its therapeutic potential in melanoma. The level of expression of these two proteins was determined by western blotting for proteins in the SK-MEL-28 and LU-1205 cells after 24 h of treatment with Lebein (Figure 5A,B). The results revealed a reduction in BCL-2 protein levels in both melanoma cell lines in parallel with increasing amounts of BAX.

The BH3-only proteins Bim and Bmf are immediate upstream triggers for Bax activation. We investigated which BH3-only proteins were responsible for Lebein-induced activation of Bax. Thus, Lebein treatment significantly increased the protein expression of Bim (Figure 5A) without affecting the expression of other BH3-only proteins such as Bmf in melanoma cells. Consequently, we concluded that Lebein-induced apoptosis responses are mediated by Bim and by dysregulation of the BAX/BCL-2 ratio.

Western blot experiments on well-known proteins induced during apoptosis were developed to confirm the activation and nature of the apoptotic signals triggered by Lebein in melanoma cells. Because caspases are often associated with apoptosis [37], we explored caspase-3, caspase-8 and PARP cleavage protein levels after Lebein treatment of SK-MEL-28 and LU-1205 cells (Figure 5C). Interestingly, our western blot results showed no activation or changes in protein levels, suggesting Lebein-activation of a caspase-independent apoptosis pathway.

### 2.7. Lebein Induces AIF-Mediated Apoptosis in Melanoma Cells

**Figure 6:**
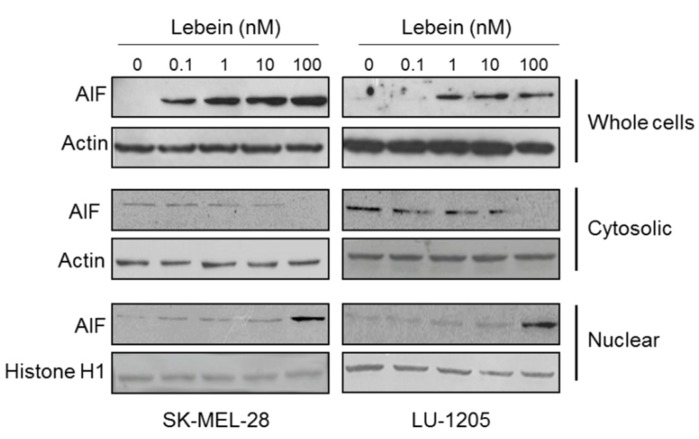
Lebein induces expression of AIF in melanoma cells. SK-MEL-28 and LU-1205 cells were treated with indicated Lebein concentrations for 24 h. AIF protein levels in whole cell lysates, nuclear and cytoplasmic extracts were detected by western blot using specific antibodies. Individual experiments were performed in triplicate.

The use of caspase inhibitors in many experiments showed that cells could die displaying a similar morphology to apoptotic cells independently of caspase activation [38]. Numerous studies have linked this phenomenon to the release of apoptosis inducing factor (AIF) from the mitochondria, followed by its translocation into the nucleus mediated by a nuclear localization signal [39,40]. After determining that Lebein induces apoptosis in both melanoma cell lines through a caspase-independent pathway, we explored the possibility of AIF activation by Lebein. After 24 h of treatment, we observed an increase in AIF protein expression in a dose dependent manner in whole cell lysates (Figure 6), with a very pronounced increase in AIF levels, especially at 10 and 100 nM doses. Also, we found that in Lebein-treated cells the level of AIF decreased in the cytoplasm and increased in the nucleus. However, we could not detect the release of cytochrome c after Lebein treatment (data not shown). The results indicate that AIF translocates to the nucleus and induces cell death via caspase-independent pathway. In summary, our results showed that Lebein could be considered as a promising candidate for melanoma tumour prevention by inducing cell apoptosis through activation of AIF and the caspase-independent cell death program.

## 3. Discussion

Melanoma is a very aggressive form of skin cancer with an increasing incidence in the population. The two major problems of melanoma are its high metastatic capacity and its resistance to conventional therapies, such as chemo- and radiotherapies that are used for other types of cancer. In recent years, there have been improvements in melanoma therapy with the emergence of BRAF-MEK inhibitors and immunotherapy [3]. However, these treatments are not completely successful, and there are different resistance mechanisms that lead to recurrence and toxic side effects. Thus, it is necessary to explore new therapeutic strategies.

The introduction of new natural compounds for cancer treatment in clinics has motivated the use of different molecules isolated from snake venoms as disintegrins. These are small peptides that are able to specifically bind to cellular integrins acting as receptor antagonists by blocking integrin’s activity, which is important for cell-cell and cell-matrix adhesion, proliferation, angiogenesis and invasiveness [9]. Herein, we determined whether Lebein an RGD heterodimeric disintegrin protein isolated in our lab from *Macrovipera lebetina* snake venom was able to affect melanoma cell viability. MITF is the master regulator of melanoma cells, and it is responsible for controlling most of the important physiological cellular processes, such as proliferation, invasiveness, stemness, drug resistance and other functions. It has been established that increasing cellular levels of MITF are responsible for a melanoma phenotype switching from a dormant state with low levels of MITF to a proliferative state with intermediate MITF levels and finally to a differentiated phenotype with higher MITF levels. MITF regulation is complex and, at transcriptional levels, is regulated by LEF1/TCF β-catenin, SOX10, PAX3 and CREB as positive regulators and the transcription factors BRN2 and GLI2 as negative transcriptional regulators [41]. In addition, MITF is also regulated by posttranslational modifications such as acetylation, sumoylation and phosphorylation [42]. Different studies have also established that MITF intracellular levels are negatively regulated by ERK phosphorylation, leading to proteosomal degradation. Our results suggested that Lebein induced inhibition in melanoma cellular proliferation that corresponded to MITF overexpression through decreases in ERK phosphorylation. We cannot claim that this is the only mechanism by which MITF is up-regulated because, as we mentioned above, there are other possible regulation pathways. Lebein treatment induced an increase in MITF corresponding to a phenotype switch from proliferative to differentiated and less invasive with E-cadherin overexpression. After Lebein exposure, cells changed their morphology, becoming more detached and with an appearance usually associated with cell death.

Under physiological conditions, cancer cells maintain ROS levels in equilibrium to prevent cell damage. Detoxification of ROS is mediated by antioxidant enzymes or by molecules such as flavonoids, glutathione and vitamins A, C and E that specifically scavenge different types of ROS. Different chemotherapeutic strategies are directed to increase cellular ROS levels triggering irreparable damages and tumour cell apoptosis [43]. However, some studies have indicated that elevated levels of intracellular ROS contribute to early events involved in cancer initiation and progression. For this reason, these reports suggested the use of antioxidants to reduce ROS levels in tumour cells [16]. We propose Lebein as a treatment that acts as a pro-oxidant on LU-1205 cells in which intracellular levels of ROS do not reach a cell toxicity level to produce apoptosis. On the contrary, in SK-MEL-28 cells, Lebein acts as an antioxidant to deplete ROS from tumour cells.

One of the most studied forms of cell death is apoptosis, which is characterized by, among other features, chromosomal DNA fragmentation. This is a tightly regulated process that can be activated in different ways due to cellular stress or in response to signals from other cells. One of the key signals for activation of apoptosis is the increase in the proapoptotic protein BAX and the decrease in the antiapoptotic protein BCL-2. Once this is triggered, there is a release of cytochrome c from the mitochondria that usually activates caspases. However, there are some cases of caspase-independent apoptosis in which there is no caspase activation, but release of AIF from the mitochondria, nuclear translocation and DNA fragmentation occur. Our results suggested that Lebein induces an apoptotic cell death signal, with morphological cell changes such as rounding and blebbing. There is an increase in Bim expression and the BAX/BLC-2 ratio but no increase in the expression or activation of the caspase pathway. Lebein was able to induce caspase-independent apoptosis with accumulation of AIF.

## 4. Conclusions

In summary, we propose Lebein as a new potential active compound that can be used against melanoma cells. Lebein is able to modify intracellular levels of MITF, imposing a change in the phenotype of melanoma cells from proliferative to differentiated and noninvasive. Further, Lebein induced changes in melanoma cell ROS generation and p53 levels, with both depending on the cell lines and the p53 mutation status. Importantly, it activates an apoptotic caspase-independent program that contributes to the elimination of cell populations. Although it is necessary to perform more specific in vivo experiments using animal models, we suggest that Lebein is a good candidate as a natural compound for further studies as a potential anti-melanoma therapy on its own or in combination with other more established drugs. Nevertheless, to obtain more detailed insight into the mechanisms of Lebein activity, future research is warranted and should focus on the expression of proteins and enzyme activities, considering that Lebein could be a potential therapeutic agent against melanoma and that it can also modulates oxidative stress and induces DNA damage in tumour cells.

## 5. Materials and Methods

### 5.1. Cells and Reagents

Lebein from venom of the snake *Macrovipera lebetina* was purified as described previously [44]. SK-MEL-28 and LU-1205 melanoma cancer cell lines were purchased from the American Type Culture Collection (ATCC), and they were routinely authenticated by genotype profiling according to ATCC guidelines. Cells were routinely tested for mycoplasma contamination using a MycoSETTM Mycoplasma Real-Time PCR detection Kit (Life Technologies, Foster City, CA, USA). Cells were maintained in the appropriate culture medium; i.e., Dulbecco’s modified Eagle’s medium (Invitrogen, Barcelona, Spain) supplemented with 10% foetal bovine serum (FBS) and antibiotics. Cells were plated and allowed to adhere overnight in the culture medium before treatments. Antibodies against the following proteins were used for western blotting. MITF was obtained from Millipore (Madrid, Spain) and β-actin and phospho-ERK were obtained from Sigma (L’Isle d’Abeau, Chesnes, France). Cleaved PARP (Cl-PARP), PARP, Caspase 3, Caspase 8, E-cadherin, p53, Phospho p53 (Ser-15), Histone H1, BAX, BCL-2, Bim, Bmf and AIF were obtained from Cell Signaling Technology (Danvers, MA, USA).

### 5.2. Cell Viability and Apoptosis Assays

Cell viability was evaluated by a colorimetric assay for mitochondrial function using the 3-(4,5-dimethylthiazol-2-yl)-2,5-diphenyl-tetrazolium bromide (MTT, Sigma-Aldrich, St. Louis, MO, USA) cell proliferation assay. For this assay, cells were plated in a 96-well plate at a density of 1000–2000 cells/well. Apoptosis was analysed using ELISA (Cell Death Detection ELISAPLUS, Roche Diagnostics, Barcelona, Spain) to detect mono- and oligonucleosomes in the cytoplasmic fractions of cell lysates using biotinylated anti-histone and peroxidase-coupled anti-DNA antibodies. The amount of nucleosomes was photometrically quantified at 405 nm by determining the peroxidase activity that was retained in the immunocomplexes. Apoptosis was defined as the specific enrichment of mono- and oligonucleosomes in the cytoplasm and was calculated by dividing the absorbance of the treated samples by the absorbance of the untreated samples after correcting for the number of cells. The induction of apoptosis in each melanoma cell line after 7 h of treatment with 2 µM staurosporin (100% apoptotic cells) was used to calculate the number of apoptotic cells. Apoptosis was also assessed by the PE Annexin-V apoptosis detection kit (BD Biosciences, San Jose, CA, USA) according to the manufacturer’s protocol. For caspase inhibitor activity assay, cells were pre-incubated with a pan-caspase inhibitor, Z-VAD-FMK (20 µM) (BD Biosciences, San Jose, CA, USA) for 2 h before Lebein-treatment. Stained cells were analyzed on a BD FACScanto II flow cytometer (BD Biosciences, San Jose, CA, USA) and further analyzed with BD FACSDiva 6 software (BD Biosciences, San Jose, CA, USA). Cell death was quantitatively evaluated by measuring the proportion of annexin positive cells, regardless of their staining for 7-AAD in order to include both apoptotic and necrotic cell death. Values are given in percent of total cell number. Percentage of apoptotic cells (%) was calculated as follows: Early apoptotic cells (%) + late apoptotic cells (%).

### 5.3. Evaluation of ROS Generation

A cell-permeable fluorogenic probe, CM-H2DCFDA, provided by Life Technologies (Thermo Fisher Scientific, Waltham, MD, USA), was used to evaluate ROS generation. Passively, this molecule diffuses into cells, where its thiol-reactive chloromethyl group reacts with intracellular glutathione and other thiols, and its acetate groups are cleaved by intracellular esterases. Subsequent oxidation leads to a fluorescent adduct in the presence of ROS. SK-MEL-28 and LU-1205 cells were seeded in 96-well plates (2000 cells/well) and treated for 24 h with different concentrations (0.1–100 nM) of Lebein. After washing with PBS, cells were incubated with CM-H2DCFDA (10 µM) at 37 °C for 30 min in the dark. Fluorescence was detected with excitation and emission wavelengths of 492 and 517 nm, respectively.

### 5.4. Cell Fractionation

Cells were collected by centrifugation and washed in ice-cold PBS. Cell pellets were resuspended in buffer 1 containing 25 mM HEPES, pH 7.9, 5 mM KCl, 0.5 mM MgCl_2_, 1 mM DTT supplemented with protease inhibitors (Roche Diagnostics, Barcelona, Spain). Thereafter, NP-40 was added at a final concentration of 1% (*v*/*v*) and lysates were incubated for 15 min on ice. Nuclei were pelleted by centrifugation at 2500 rpm for 1 min at 4 °C and supernatant containing cytoplasmic proteins was collected and stored at −30 °C. Remaining nuclei were washed in buffer 1 containing NP-40 (1%). The nucleic pellets were lysed in buffer 2 containing 25 mM HEPES pH 7.9, 350 mM NaCl, 10% Sucrose, 0.05% NP-40, 1 mM DTT and Protease Inhibitors. Nucleic lysates were rotated for 1 h at 4 °C. Supernatants containing soluble nucleic proteins were collected by centrifugation at 14,000 rpm for 10 min and stored at −30 °C.

### 5.5. Western Blotting Analysis

Cells were lysed in Laemmli buffer after being treated with different concentrations of Lebein. Whole cell lysates (30 µg/lane), nuclear and cytosolic extracts (25 µg/lane) were separated by SDS-PAGE in 10% polyacrylamide gels and were then transferred onto a polyvinylidene difluoride membrane (Immobilon-P, Millipore, Madrid, Spain). Immunoreactive proteins were detected by an enhanced chemiluminescence detection system (ECL, Pierce, Rockford, IL, USA).

### 5.6. Real Time Quantitative RT-PCR

qRT-PCR in a 96-well format (Applied Biosystems 7500, Applied Biosystems, Foster City, CA, USA) was used to evaluate gene expression profiles. mRNA extraction and cDNA synthesis were performed as described previously [45]. The following primers for human genes were used: β-actin (forward: 5′-AGA AAA TCT GGC ACC ACA CC-3′ reverse: 5′-GGG GTG TTG AAG GTC TCA AA-3′); BAX (forward: 5′-TTT GCT TCA GGG TTT CAT CC-3′; reverse: 5′-GCC ACT CGG AAA AAG ACC TC-3′); BCL-2 (forward: 5′-GGA TTG TGG CCT TCT TTG AG-3′; reverse:5′-CCA AAC TGA GCA GAG TCT TC-3′); E-cadherin (forward: 5′-TCC ATT TCT TGG TCT ACG CC-3′; reverse: 5′-CAC CTT CAG CCA TCC TGT TT-3′); and MITF (forward: 5′-GCG CAA AAG AAC TTG AAA AC-3′; reverse: 5′-CGT GGA TGG AAT AAG GGA AA-3′). Primer Express version 2.0 software (Applied Biosystems) was used to design the primers provided by Life Technologies.

### 5.7. Statistical Analysis

Graph-pad prism was used to evaluate the results as the mean ± SD values from three to five determinations in triplicate. Data were analysed using one-way ANOVA or two-way ANOVA. Differences were considered to be statistically significant at *p* < 0.05.

## Abbreviations

The following abbreviations are used in this manuscript:

AIF	apoptosis inducing factor
ATCC	american type culture collection
BAX	BCL-2-associated X protein
BCL-2	B-cell lymphoma-2
CM-H2DCFDA	5-(and-6)-chloromethyl-2′,7′-dichlorodihydrofluorescein diacetate
ELISA	enzyme-linked immunosorbent assay
ERK	extracellular signal-regulated kinase
MITF	microphthalmia-associated transcription factor
MTT	3-(4,5-dimethylthiazol-2-yl)-2,5-diphenyl-tetrazolium bromide
NAC	N-acetylcysteine
PARP	poly-ADP-ribose polymerase
PD-1	programmed death 1
PD-L1	programmed death-ligand 1
ROS	reactive oxygen species
qRT-PCR	quantitative reverse-transcription-polymerase chain reaction
SDS-PAGE	sodium dodecyl sulfate polyacrylamide gel electrophoresis
